# Community-based educational intervention to limit the dissemination of community-associated methicillin-resistant *Staphylococcus aureus *in Northern Saskatchewan, Canada

**DOI:** 10.1186/1471-2458-12-15

**Published:** 2012-01-06

**Authors:** George R Golding, Brian Quinn, Kirsten Bergstrom, Donna Stockdale, Shirley Woods, Mandiangu Nsungu, Barb Brooke, Paul N Levett, Greg Horsman, Ryan McDonald, Brian Szklarczuk, Steve Silcox, Shirley Paton, Mary Carson, Michael R Mulvey, James Irvine

**Affiliations:** 1 National Microbiology Laboratory, Winnipeg, MB, Canada; 2 Population Health Unit, LaRonge, SK, Canada; 3 University of Manitoba, Winnipeg, MB, Canada; 4 Northern Intertribal Health Authority, Prince Albert, SK, Canada; 5 Red Earth First Nation, Meadow Lake, SK, Canada; 6 Saskatchewan Disease Control Laboratory, Regina, SK, Canada; 7 Public Health Agency of Canada, Ottawa, ON, Canada; 8 Do Bugs Need Drugs, Edmonton, AB, Canada; 9 Population Health Unit, Athabasca, Keewatin Yatthé and Mamawetan Churchill River Health Authorities, 2nd Floor, Lac La Ronge Indian Band Office, Box 6000, LaRonge, SK S0J 1L0, Canada

## Abstract

**Background:**

Surveillance examining the incidence of community-associated methicillin-resistant *Staphylococcus aureus *(CA-MRSA) was conducted over 8 years beginning in 2001 in three health regions covering the northern half of Saskatchewan. The annual rate of individuals reported with CA-MRSA infection in these regions dramatically increased from 8.2 per 10,000 population in 2001 (range to 4.4-10.1 per 10,000) to 168.1 per 10,000 in 2006 (range 43.4-230.9 per 10,000). To address this issue, a team of community members, healthcare professionals, educators and research scientists formed a team called "the Northern Antibiotic Resistance Partnership" (NARP) to develop physician, patient, community, and school based educational materials in an attempt to limit the spread of CA-MRSA.

**Methods:**

Posters, radio broadcasts, community slide presentations, physician treatment algorithms, patient pamphlets, and school educational programs Do Bugs Need Drugs http://www.dobugsneeddrugs.org and Germs Away http://www.germsaway.ca were provided to targeted northern communities experiencing high rates of infections.

**Results:**

Following implementation of this program, the rates of MRSA infections in the targeted communities have decreased nearly two-fold (242.8 to 129.3 infections/10,000 population) from 2006 to 2008. Through pre-and post-educational intervention surveys, this decrease in MRSA infections coincided with an increase in knowledge related to appropriate antimicrobial usage and hand washing in these communities.

**Conclusion:**

These educational materials are all freely available http://www.narp.ca and will hopefully aid in increasing awareness of the importance of proper antimicrobial usage and hygiene in diminishing the spread of *S. aureus *and other infectious diseases in other communities.

## Background

Community-associated methicillin-resistant *Staphylococcus aureus *(CA-MRSA) is an emerging pathogen in North America. These strains differ from typical healthcare associated MRSA in that they often harbour the Panton-Valentine Leukocidin (PVL) toxin, and are generally more susceptible to classes of antimicrobials other than the beta-lactam derived drugs, although increasing resistance to antimicrobials are now being reported in many PVL positive CA-MRSA isolates [1]. These CA-MRSA strains have been associated primarily with skin and soft tissue infections [2-4] with increased morbidity and mortality [5], and are now entering and being disseminated within health care facilities [6].

In the 1990s CA-MRSA had been reported only sporadically in Canada [7,8]. However, since that time two main CA-MRSA strains have emerged in Canada, CMRSA7 (similar to USA400; ST1-MRSA-IV) and CMRSA10 (similar to USA300; ST8-MRSA-IV) [2,6,9,10]. CMRSA10 has been identified predominantly in the western provinces of Canada and high incidences have been reported in the homeless, incarcerated, and intravenous drug user populations in the Calgary [11,12] and Vancouver [13] health regions. CMRSA7 was first reported in Manitoba causing an outbreak in the southern portion of that province in the late 1990's, but spread to northern regions of the province [14-16] and communities further north in Nunavut [17]. CMRSA7 was thereafter seen in central eastern [4] and northern Saskatchewan with annual crude rates of infections as high as 482 per 10,000 population in some communities [2].

Anecdotal evidence from northern Saskatchewan and other Canadian communities suggests that physicians and prescribing nurses frequently opt to empirically prescribe antimicrobials without obtaining laboratory identification of the infecting organism or drug resistance. It has therefore been speculated that liberal antimicrobial prescribing practices in northern communities may contribute to community acquisition of CA-MRSA [3]. In a previous northern Saskatchewan case control study, the average number of antimicrobials prescribed in the year prior to acquiring a CA-MRSA infection was 2.5 (median 2, range 0-10) [3]. Previous antimicrobial usage is a known risk factor for acquiring CA-MRSA [18] and a recent intervention study in children has demonstrated that decreasing unnecessary usage can decrease the prevalence of MRSA in a community setting [19]. Data obtained for the treatment of CA-MRSA infections in northern Saskatchewan communities also revealed that a high proportion of individuals (88.7%) were prescribed antimicrobials, of which 57.1% were treated with a β-lactam, which could be contributing to treatment failures, recurrent infections, and/or further dissemination of CA-MRSA within these communities [3].

In this report, we describe the rapid emergence of CA-MRSA in northern regions of Saskatchewan and the potential effect of a community-based educational intervention http://www.narp.ca to limit its spread in targeted communities.

## Methods

### Study area

The study area included three northern health regions in Saskatchewan: Athabasca Health Authority (AHA), Keewatin Yatthé Health Region (KYHR), and Mamawetan Churchill River Health Region (MCRHR) http://www.health.gov.sk.ca/health-regions-map. This 317,000 km^2 ^(122,400 miles^2^) boreal forest area includes 35,819 people (Saskatchewan Health, 2010) with 86% of Aboriginal descent (either First Nation or Métis) with 46% living in First Nation "reserve" communities (Statistics Canada). Health care services ranged from primary care nursing health centers with weekly fly-in physician clinics to a 40-bed hospital with resident physicians.

### Determination of case incidence

A MRSA case was defined as a laboratory isolation of MRSA from an infection site. Isolates from the nose or groin without specification of an infection or an unspecified screening or swab, and isolates obtained from an individual less than 2 months from previous isolates were excluded. Isolates with information associated with characteristics of healthcare-associated MRSA (HA-MRSA) were also excluded. Healthcare-associated cases included post-surgical infections, dialysis, long-term care residence, or indwelling catheter or percutaneous medical device.

### Data collection

The study was conducted over 8 years from January 1, 2001 to December 31, 2008. Laboratory identification of all MRSA cases were reported to the First Nations and regional health Medical Health Officers. Over the 8 year study period, a total of 3317 MRSA isolates were submitted from the three northern Saskatchewan health regions. Of these, 586 isolates did not meet the inclusion criteria of this study and were excluded from further analysis. The remaining 2731 isolates were identified as cases of CA-MRSA.

### Educational intervention

In an attempt to address the increasing CA-MRSA rates in these regions, a two pronged educational strategy was developed and approved by the University of Manitoba, University of Saskatchewan, and Health Canada Research Ethics Boards. The first approach involved educational activities aimed at healthcare providers, which included the development and implementation of an active surveillance system to enhance the collection of data, including microbiologic information (organism identification and susceptibility data), prescribing practices, and general patient demographic information for clients in 3 communities in northern Saskatchewan from October 2005-March 2008 [2]. Secondly, a case-control study was initiated in September 2004-March 2007 to determine the risk factors associated with CA-MRSA infections [3]. The information gained from these 2 projects were reported to physicians and healthcare providers, which included the development and distribution of local guidelines for management of suspected CA-MRSA skin and soft tissue infections (available at http://www.narp.ca). Physician offices and community health centers were also provided with information pamphlets describing MRSA for distribution.

The second approach involved addressing the antimicrobial resistance issues through educational activities aimed at the community. From a community perspective, posters describing the study, radio interviews, and slide presentations about antibiotic resistance and CA-MRSA were provided to the targeted communities. Radio broadcasts were also developed and broadcast on local radio stations in English, Cree, and Dene to further educate the general public on skin and soft tissue infections, hand washing, and completing the entire course of antibiotics. From a community perspective, pre- (2006) and post-education (2008) surveys were distributed in an attempt to capture knowledge translation of these NARP educational materials. In 2006, training sessions for an existing educational program Do Bugs Need Drugs http://www.dobugsneeddrugs.org was rolled-out into the targeted communities for children in grades K-3 (ages 5-8). To determine the potential effectiveness of the Do Bugs Need Drugs program a pocket chart was developed and utilized before and several months following the implementation of this program in the classrooms. In 2007, the Germs Away curriculum was developed for students in grades 4-6 (ages 9-11) and piloted in 19 northern Saskatchewan schools. The 7 activities in the Germs Away curriculum aimed to introduce basic concepts related to the spread of infectious diseases through contact, with learning outcomes related to health and science. The site also included desktop wallpapers, posters, hand washing posters aimed at children, and a teacher's section offering all Germs Away teaching resources. Germs Away also includes a newly developed interactive video game http://www.germsaway.ca, which was developed to supplement the Germs Away curriculum, but was not available during the intervention period. The Germs Away curriculum and game is available in English and French.

It should be noted that a couple of additional northern Saskatchewan schools outside the targeted communities had received some of the school-based educational components of the intervention in 2006 with some additional spill over to other northern communities in late 2008. All primary healthcare providers in the northern areas were also provided with the CA-MRSA treatment/management algorithms in 2006/7.

## Results and discussion

Over the 8 year study period, the annual rate of individuals reported with CA-MRSA infection in northern Saskatchewan have dramatically increased from 8.2 per 10,000 population in 2001 to 142.6 per 10,000 in 2008, which is almost 10 times higher than all reported MRSA cases in neighboring northern Manitoba health regions [14] and the province of Alberta [12]. The reporting structure is similar between Saskatchewan and Alberta, but the Manitoba rates included both colonizations/infections and did not differentiate between HA- and CA-MRSA strains, which further highlights the rapid emergence and dissemination of CA-MRSA in northern Saskatchewan.

The majority of isolates were obtained from skin and soft tissue infections (78.2%), followed by ear (6.7%), urogenital (2.4%), respiratory (1.1%), and joint/blood infections (0.4%). A total of 11.2% of the infections were unspecified. Molecular typing of a subset of 404 isolates by pulsed-field gel electrophoresis revealed CMRSA7 to be the predominant strain type (92.3%) followed by CMRSA10 (4.7%). These results are similar to those previously described in specific northern Saskatchewan communities dealing with high rates of CA-MRSA infections [2,3], suggesting clonal dissemination of CMRSA7 throughout the northern regions of the province.

To help address the rapid emergence and dissemination of CA-MRSA in northern Saskatchewan, a team of community members, healthcare professionals, educators and research scientists formed a team called NARP http://www.narp.ca and established: 1) An active surveillance program in target northern Saskatchewan communities [2]; 2) A case-control study to determine risk factors for the acquisition of CA-MRSA [3]; and 3) A community, school, and health worker educational initiative through a collaboration of federal, provincial, regional and First Nations health authorities and laboratories.

**Figure 1:**
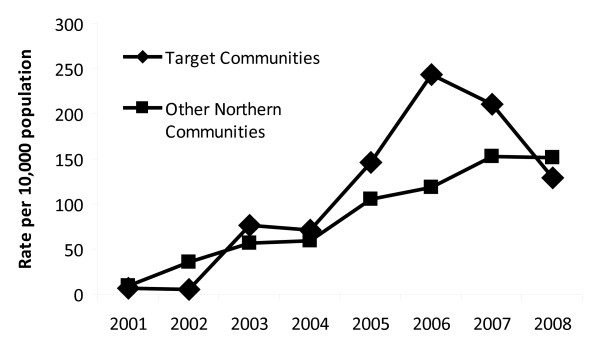
**Rates of CA-MRSA infections in NARP targeted communities following implementation of the NARP educational program, which began in 2006, in comparison to other northern communities in Saskatchewan**.

Following implementation of this educational program, the rates of MRSA infections in the targeted communities have decreased nearly two-fold (243 to 129 infections/10,000 population) from 2006 to 2008, in comparison to other non-target northern communities in Saskatchewan where the rates have continued to increase (119 to 151 infections/10,000 population) over the same time period (Figure 1). This decrease in CA-MRSA infections in the targeted communities was observed across all age groups examined and was not the result of reporting bias or a decrease in the number of laboratory tests.

It is important to note that this study was purely observational and therefore it is difficult to directly relate decreased CA-MRSA infections to the educational intervention. However, we did demonstrate positive knowledge translation of our educational materials using pre-/post-community surveys and a pocket chart to evaluate the Do Bugs Need Drugs program. The pre- (n = 94) and post-community (n = 87) surveys involved questions regarding the appropriate usage of antimicrobials, differences between bacterial and viral infections, and overall awareness of NARP. Improved responses to 19/20 questions were noted in the post-community education survey. This included for example significant improvements in responses for individuals who would request antibiotics from the doctor, or seek out another doctor, if they were not prescribed antibiotics for themselves or their children (*p *= 0.004-0.03) and the importance of not using left over antibiotics at home for new illnesses (*p *= < 0.001). The Do Bugs Need Drugs pocket chart collected data from kindergarten to grade 3 school aged children both pre- (n = 821) and post-education (n = 685) and demonstrated increased knowledge on the size of germs (*p *= < 0.001), when and how to wash your hands (*p *= < 0.001 and *p *= 0.004, respectively), and what kind of germs can be killed by antibiotics (*p *= 0.066).

## Conclusions

The Germs Away curriculum is available for free download and also includes a podcast series on how to teach the program http://www.germsaway.ca. Although not evaluated, we believe the lessons provided in the Germs Away curriculum (e.g. proper hand washing and cough etiquette) would be an additional valuable tool for public health to implement in school health curriculums for the prevention and mitigation of CA-MRSA infections, as well as other communicable diseases. For instance, meta-analysis of community-based hand washing campaigns have shown reductions in diarrheal diseases of approximately 30% [20] as well as some additional evidence of reducing respiratory disease [21,22]. Additional community-based NARP educational materials http://www.narp.ca (e.g. treatment algorithms, surveillance protocols, radio ads, posters, patient pamphlets, and community presentation slide decks) are also freely available http://www.narp.ca and will hopefully aid in increasing awareness of the importance of proper antimicrobial usage and hygiene in limiting the spread of CA-MRSA disease in other communities. Well-conducted rigorous trials are warranted in future study to determine the direct effectiveness of this entire educational intervention strategy.

## Competing interests

The authors declare that they have no competing interests.

## Authors' contributions

GG, MM, and JI drafted the manuscript. DS, SW, MN, BB, PL, RM, GH, SP, MC, MM, GG, and JI participated in the design and implementation of the study. KB was the NARP education coordinator and trainer. MC provided assistance in rolling out the Do Bugs Need Drugs program. BS and SS were the technical leads, and MM, KB, BB, GG, JI, SP, DS, and SW participated, in the development of the Germs Away curriculum. BQ, GG, KB, and JI collected and analyzed the data. All authors have read and approved the final manuscript.

## Pre-publication history

The pre-publication history for this paper can be accessed here:

http://www.biomedcentral.com/1471-2458/12/15/prepub
